# Supplementary material for: The Systems Biology Research Tool: evolvable open-source software
Source: BMC Syst Biol. 2008 Jun 29;2:55. doi: 10.1186/1752-0509-2-55 (PMC2446383; doi:10.1186/1752-0509-2-55)
Supplement: Additional file 1 — SBRT Archive. An archive of the current version of the Systems Biology Research Tool. [file 1752-0509-2-55-S1.zip › sbrt-1.4.0/doc/developers_guide/api/sbrt/shell/text/fba/package-frame.html]

sbrt.shell.text.fba


sbrt.shell.text.fba

Interfaces 
  
*CatalystListFormat*
  
*CatalystSetFormat*
  
*ConstraintsFileLineFormat*
  
*FbaOptFileLineFormat*
  
*FbaOptHeaderFormatter*
  
*FbaOptHeaderParser*
  
*FluxCapFormat*
  
*FluxomeSolutionFileLineFormat*
  
*FluxVectorFormat*
  
*RxnNameExprFormat*
  
*RxnNameListFormat*
  
*RxnNameOrExprFormat*
  
*RxnNameSetFormat*

Classes 
  
BiggBracketParserV1
  
BiggRxnNodeParser
  
CatalystListFormatV1
  
CatalystSetFormatV1
  
CatalystVerifier
  
ChemSpeciesVerifier
  
ConstraintsFileLineFormatV1
  
ConstraintsFileLineFormatV2
  
FbaOptFileLineFormatV1
  
FbaOptHeaderFormatterV1
  
FbaOptHeaderParserV1
  
FluxCapFormatV1
  
FluxomeSolFileLineFormatV1
  
FluxVectorFormatV1
  
IrfFormatV1
  
IrrevRxnFormatV1
  
PalssonRxnNodeParser
  
RxnNameExprFormatV1
  
RxnNameListFormatV1
  
RxnNameOrExprFormatV1
  
RxnNameSetFormatV1
  
RxnNameVerifier
